# Identification of Genetic Loci Affecting the Severity of Symptoms of Hirschsprung Disease in Rats Carrying *Ednrb^sl^* Mutations by Quantitative Trait Locus Analysis

**DOI:** 10.1371/journal.pone.0122068

**Published:** 2015-03-19

**Authors:** Jieping Huang, Ruihua Dang, Daisuke Torigoe, Chuzhao Lei, Xianyong Lan, Hong Chen, Nobuya Sasaki, Jinxi Wang, Takashi Agui

**Affiliations:** 1 College of Animal Science and Technology, Northwest A&F University, Yangling, Shaanxi, China; 2 Laboratory of Laboratory Animal Science and Medicine, Department of Disease Control, Graduate School of Veterinary Medicine, Hokkaido University, Hokkaido, Japan; 3 Laboratory of Laboratory Animal Science and Medicine, School of Veterinary Medicine, Kitasato University, Aomori, Japan; National Institute of Genetics, JAPAN

## Abstract

Hirschsprung’s disease (HSCR) is a congenital disease in neonates characterized by the absence of the enteric ganglia in a variable length of the distal colon. This disease results from multiple genetic interactions that modulate the ability of enteric neural crest cells to populate developing gut. We previously reported that three rat strains with different backgrounds (susceptible AGH-*Ednrb^sl/sl^*, resistant F344-*Ednrb^sl/sl^*, and LEH-*Ednrb^sl/sl^*) but the same null mutation of *Ednrb* show varying severity degrees of aganglionosis. This finding suggests that strain-specific genetic factors affect the severity of HSCR. Consistent with this finding, a quantitative trait locus (QTL) for the severity of HSCR on chromosome (Chr) 2 was identified using an F_2_ intercross between AGH and F344 strains. In the present study, we performed QTL analysis using an F_2_ intercross between the susceptible AGH and resistant LEH strains to identify the modifier/resistant loci for HSCR in *Ednrb*-deficient rats. A significant locus affecting the severity of HSCR was also detected within the Chr 2 region. These findings strongly suggest that a modifier gene of aganglionosis exists on Chr 2. In addition, two potentially causative SNPs (or mutations) were detected upstream of a known HSCR susceptibility gene, *Gdnf*. These SNPs were possibly responsible for the varied length of gut affected by aganglionosis.

## Introduction

Hirschsprung’s disease (HSCR) or aganglionic megacolon is a neonatal intestinal obstruction syndrome characterized by the absence of the enteric ganglia along a variable length of the hindgut; this disease results in the loss of normal intestinal motility, the failure to pass meconium, and the massive distention of the intestine [1]. HSCR is classified into three types on the basis of the length of the affected segment: short-segment (80%), long-segment (15%), and total colonic aganglionosis (5%) [2]. This congenital disease occurs in 1 out of 5000 infants and is common among Asians [3]. Most HSCR cases are associated with mutations in the *RET* proto-oncogene, endothelin receptor B (*EDNRB*) gene, and glial cell line-derived neurotrophic factor (*GDNF*) gene [4]. Genes implicated in HSCR include *SOX10* [5, 6], *NRTN* [7], *ECE* [8], *ZFHXIB* [9], *PHOX2B* [10], *KIAA1279* [11], and *TCF4* [8]. However, only 20% of HSCR cases are attributed to mutations in these genes; therefore, other susceptible genes possibly exist [12]. HSCR commonly shows variable phenotypes and penetrance by familial, gender incidence, associated diseases, and aganglionosis severity. However, even familial cases characterized by the same mutation between individual family members show a large discrepancy in penetrance and the length of the influenced gut [13]. These lines of evidence imply that multiple genetic interactions modulate the development of enteric ganglia derived from neural crest cells and thus affect the final phenotype of HSCR. The current data indicate that interactions between *RET* and *EDNRB* [14, 15], *EDNRB* and *SOX10* [16, 17], and *RET* and *SOX10* [18] modulate neural crest cells during early embryonic development.

Completely homozygous deficient mutations in *EDNRB* result in the severe aganglionosis phenotype of HSCR in mice [19, 20]. Spotting lethal (*sl*) is a spontaneous null mutation with a 301 bp deletion in the rat *Ednrb* that leads to the dysfunction of the corresponding protein [21]. In our previous study, we established three rat strains that carry the *sl* mutation: AGH-*Ednrb* ^*sl*^, LEH-*Ednrb* ^*sl*^, and F344-*Ednrb* ^*sl*^ [22]. Aganglionosis in all pups of AGH-*Ednrb* ^*sl/sl*^ rats extends beyond the cecum, whereas that in pups of LEH-*Ednrb* ^*sl/sl*^ rats is confined to the middle colon. F344-*Ednrb* ^*sl/sl*^ rats display minimal (i.e., very short segment near the anus is affected) or no aganglionosis. These lines of evidence suggest that modifier genes within the genetic backgrounds of these strains significantly modulate the severity of aganglionosis. We also previously identified a significant quantitative trait locus (QTL) on chromosome (Chr) 2 using an F_2_ intercross of AGH-*Ednrb* ^*+/sl*^ and F344-*Ednrb* ^+/*sl*^ rats [23]. Thus, we hypothesized that different genetic backgrounds contain different modifiers that interact with primary mutation. We believe that these modifiers influence the development of the enteric nervous system and the variable penetrance and severity of HSCR. The present study explores the variation of aganglionosis between AGH-*Ednrb* ^*+/sl*^ and LEH-*Ednrb* ^*+/sl*^ strains to identify modifiers that may interact with the *sl* mutation of the *Ednrb* gene and influence aganglionosis in a variable length of the distal gastrointestinal tract.

## Results

### Evaluation of the aganglionosis phenotype in F_2_ intercross

The homozygous *sl* mutation of *Ednrb* in rats results in the aganglionosis phenotype. We previously found that introgression of the null mutation into the LEH strain modifies the phenotype of aganglionosis [22]. AGH-*Ednrb* ^*sl/sl*^ rats show unnatural dilation of the intestines at 14 d postnatal because of the absence of ganglion cells in the gut, starting from the anus and extending to the cecum. Compared with AGH-*Ednrb* ^*sl/sl*^ rats, LEH-*Ednrb* ^*sl/sl*^ pups at 14 d postnatal show much shorter length of enlarged intestines. The variation in the expressivity of HSCR between AGH and LEH strains results from the length of aganglionosis, as identified using whole-mount acetylcholinesterse (AChE) staining [22]. F_2_ (AGH × LEH) *Ednrb* ^*sl/sl*^ progenies with different phenotypes were established by heterozygotes. Homozygous *Ednrb* ^*sl/sl*^ animals (n = 149) were selected from F_2_ intercross on the basis of coat color [22]. The extent of the absence of ganglion cells in the gut of *Ednrb* ^*sl/sl*^ rats was assessed using microscopic examination with AChE staining. Then, the length of the gut exhibiting aganglionosis was divided by the total length of the large intestine. This ratio was used as a quantitative trait index for the QTL analysis of aganglionosis severity.

**Fig 1:**
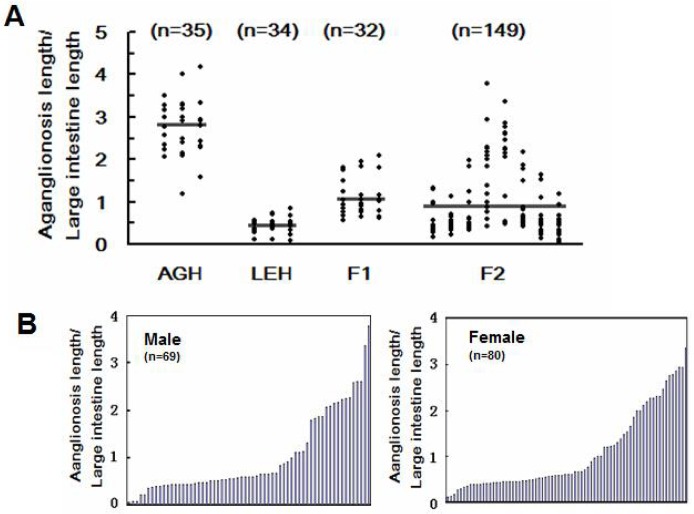
Range of aganglionosis extent. (A) Range of aganglionosis extent in 14-day-old pups from AGH-*Ednrb* ^*sl/sl*^, LEH-*Ednrb* ^*sl/sl*^, F_1_, and F_2_. Mean values are indicated by horizontal lines. (B) Distribution of the severity of aganglionosis in male and female progenies of the F_2_ generation. The Y-axis represents the ratio of the length of the aganglionic gut to the length of the large intestine used to evaluate the severity of aganglionosis.

The range of aganglionosis extent in AGH, LEH, F_1_, and F_2_ rats is presented in a scatter plot (Fig. 1A). The ratios of AGH-*Ednrb* ^*sl/sl*^ and LEH-*Ednrb* ^*sl/sl*^ rats fall on one of the two extreme values. Those of F_1_ progenies (0.8 in the mean ratio of aganglionosis) are distributed between the mean ratios of AGH (2.78 in the ratio of aganglionosis) and LEH (0.449 in the ratio of aganglionosis). The ratios among the F_2_ intercross are fairly scattered from the maximum to the minimum with the mean ratio of 0.846.

In Fig. 1B, individual traits of the male and female F_2_ intercross are arranged in accordance with the ratio of aganglionosis extent. The distribution of trait values in both males and females is consistent with the idea that the variation in aganglionosis in this population is a polygenic trait. In addition, the results showed no gender bias.

### QTL analysis for modifiers of aganglionosis severity in the F_2_ intercross of *Ednrb* ^*sl/sl*^

**Fig 2:**
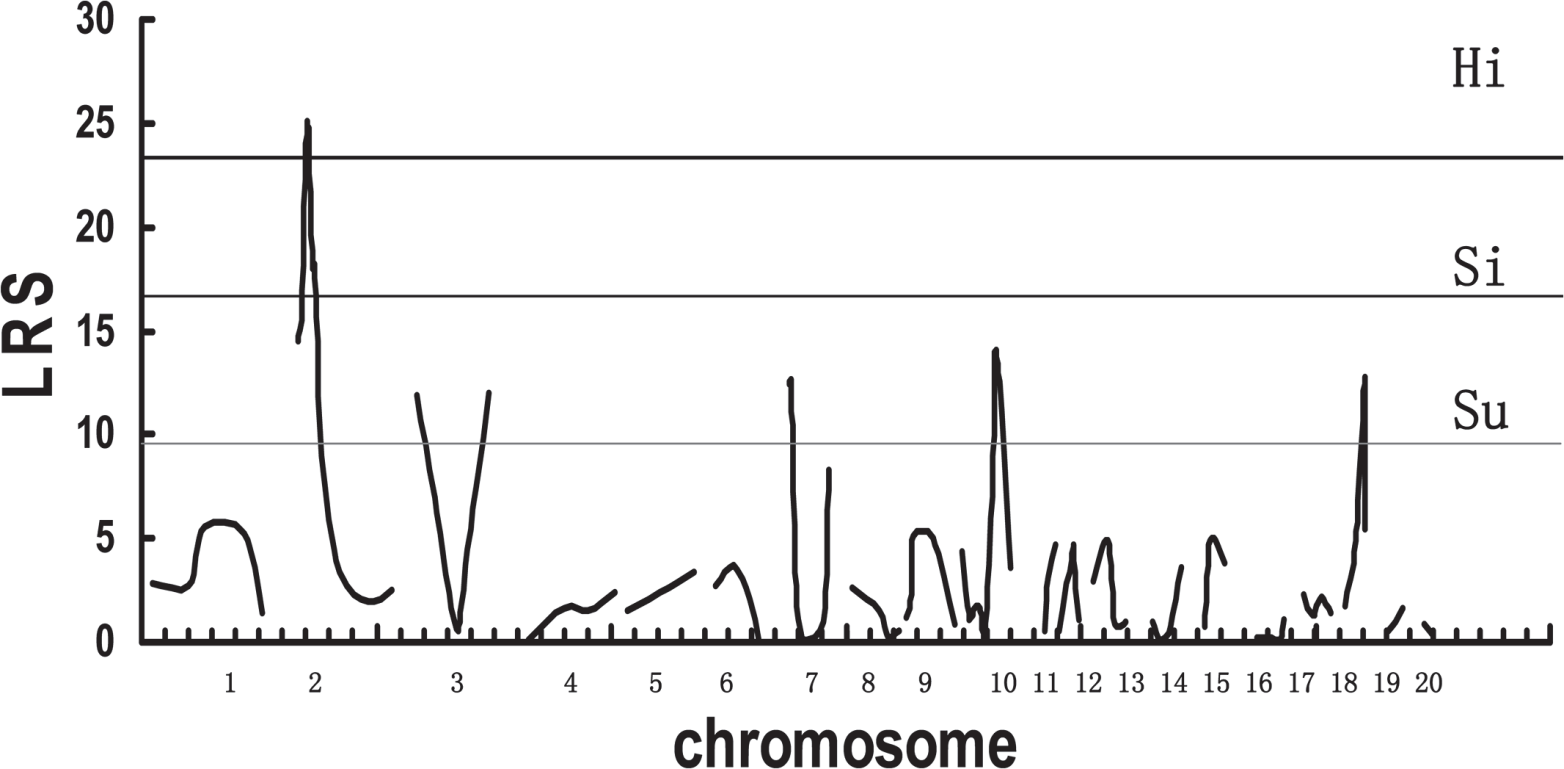
Interval mapping scans by MapManager QTXb20 in F_2_ intercross. Linkage analyses of aganglionosis severity in F_2_ intercross were performed using MapManager QTXb20 software. Recombination frequencies (%) were converted into genetic distance (centiMorgan; cM) using the Kosambi map function. Linkage data are provided as likelihood ratio statistic (LRS) scores. Genome-wide significance thresholds were calculated in terms of LRS by 1000 times permutation tests. The thresholds for suggestive (Su, LRS = 9.6), significant (Si, LRS = 17.1), and highly significant (Hi, LRS = 23) linkages are indicated in dotted, thin, and thick lines, respectively, as calculated by 1000 times permutation tests, respectively.

**Fig 3:**
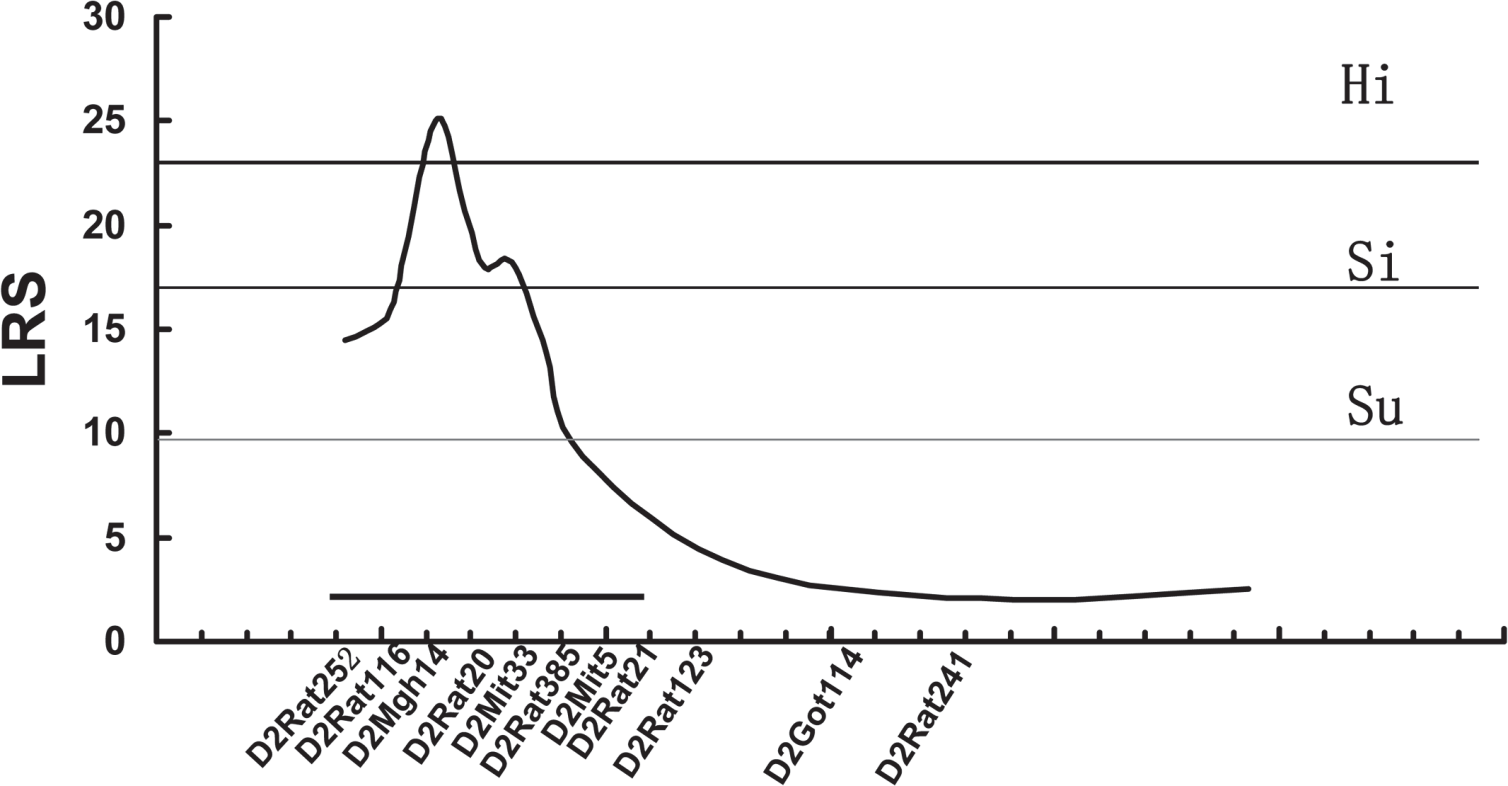
Details of suggestive and significant linkages in QTL analysis of the severity of aganglionosis. The QTL on chromosome 2 showed a significant linkage to aganglionosis severity, respectively. The dotted, thin, and thick lines represent Su, Si, and Hi thresholds, calculated by 1000 times permutation tests, respectively. The microsatellite markers used for determining genotypes of F_2_ intercross are presented along the X-axis. The gray bars on the graph indicate approximately 95% confidence intervals.

QTL analysis was carried in the F_2_ intercross. The threshold likelihood ratio statistics (LRS) for mapping was generated by MapManager QTXb20 software. In this analysis, the threshold values of suggestive, significant, and highly significant linkages were 9.6, 17.1, and 23, respectively, as calculated by 1000 times permutation tests (Fig. 2). A QTL significantly associated with aganglionosis was detected in the region around *D2Mgh14* on Chr 2 with the maximum LRS score of 25.0 (Fig. 3), which accounted for 15% of the total variance (Table 1). This result implies that the locus at the *D2Mgh14* (62 Mbp, RGSC Genome Assembly v5.0) position has a significant linkage to the severity of aganglionosis (LRS > 23).

**Table 1:** Quantitative trait loci with LRS scores (> 4.0) detected by marker regression analysis.

Chr	Locus	LRS	Contribution (%)	*P* value	CI	Additive effect
Chr 2	D2Rat252	14.5	9	0.00072	38	0.30
Chr 2	D2Rat116	16.0	10	0.00033	35	0.36
Chr 2	D2Mgh14	25.0	15	0.00000	23	0.43
Chr 2	D2Rat201	20.4	13	0.00004	28	0.40
Chr 2	D2Mit33	18.0	11	0.00012	31	0.40
Chr 2	D2Rat385	18.8	12	0.00008	30	0.41
Chr 2	D2Mit5	14.5	9	0.00073	38	0.36
Chr 2	D2Rat21	8.9	6	0.01165	61	0.29
Chr 3	D3Rat57	11.9	8	0.00260	46	‒0.24
Chr 3	D3Mgh7	7.0	5	0.03068	78	‒0.17
Chr 3	D3Rat78	12.3	8	0.00209	45	0.24
Chr 7	D7Rat31	12.4	8	0.00200	44	0.33
Chr 7	D7Got23	12.7	8	0.00176	44	0.34
Chr 7	D7Rat131	8.3	5	0.01548	65	‒0.17
Chr 10	D10Mit2	8.0	5	0.01833	68	0.15
Chr 10	D10Rat154	14.0	9	0.00091	40	0.18
Chr 18	D18Got63	12.8	8	0.00162	43	0.07

Chr, chromosome; CI, confidence interval.

### Identification of candidate genes in Chr 2

We performed a bioinformatics search combining PosMed search using NCBI (http://www.ncbi.nlm.nih.gov/probe) and PosMed (https://database.riken.jp/PosMed/) to identify biologically relevant genes from the identified QTL [24]. We used the positions (from 23 Mbp to 75 Mbp, RGSC Genome Assembly v3.4) of the markers (*D2Rat252* and *D2Rat21*), between which is the confidence interval (95% confidence level), to define the boundaries of this interval on the rat genome assembly. The keywords “aganglionosis,” “intestine,” and “neural crest cell” were used to search for genes on the rat genome. Then, the candidate genes were narrowed on the basis of their known functions and expression as listed in the PosMed database and related literature. *Gdnf*, *Ptger4*, and *Slc45a2* were identified as highly relevant candidates (Table 2). *Gdnf*, a well-known gene associated with Hirschsprung disease, plays an important role in the development of the enteric nervous system. *Ptger4*, which is related to colitis and hearing loss, is also expressed in intestine tissue [25, 26]. Disorder of cochlear cells, in which melanocytes are derived from neural crest cells, can cause hearing loss. *Slc45a2* is involved in developmental pigmentation and associated with oculocutaneous albinism [27]. The abnormal development of melanocytes drives albinism; hence, we consider *Slc45a2* as a candidate gene on the basis of its involvement with other aspects of neural crest development.

**Table 2:** List of candidate genes for the QTL associated with aganglionosis.

Gene symbol	Gene description	Mbp
*Ptger4*	Prostaglandin E receptor 4	73.9
*Gdnf*	Glial cell derived neurotrophic factor	76.9
*Slc45a2*	Solute carrier family 45, member 2	83.7

### Polymorphism analysis of candidate genes

**Fig 4:**
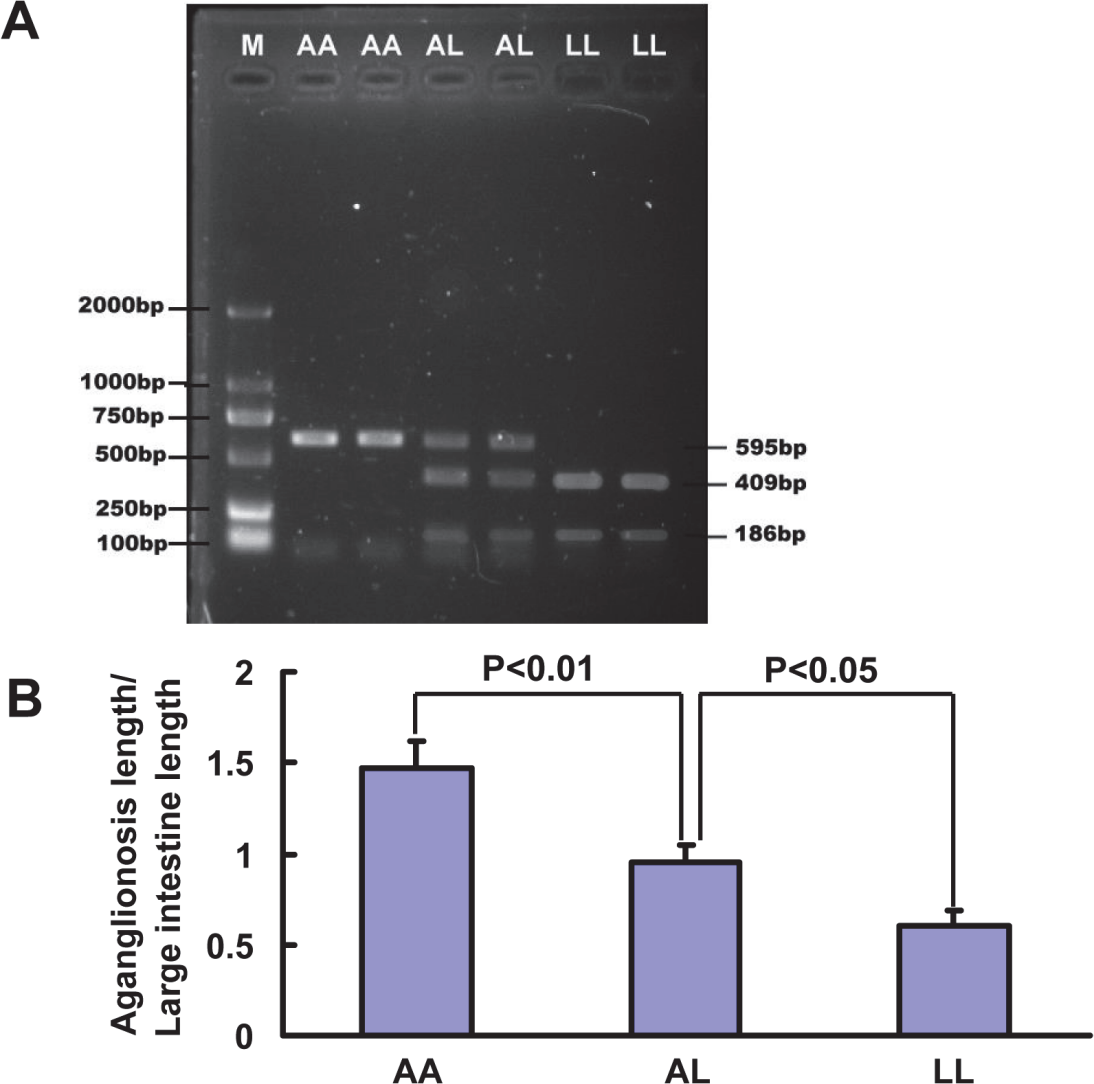
Correlation analysis of the g.76897291C > T mutation in *GDNF*. (A) Genotype groups are defined as AGH/AGH (AA), AGH/LEH (AL), and LEH/LEH (LL). AA genotype showed one band (595 bp); LL genotype showed two bands (409 and 186 bp); AL genotype showed three bands (595, 409, and 186 bp). (B) Effect of each genotype on the severity of aganglionosis. Genotyping data for the total F_2_ intercross obtained from *Sty* I were used to assess the effects of individual loci on the severity of HSCR. The mean aganglionosis severity (aganglionosis length/large intestine length) is plotted for each genotype class to show the relation of the number of AGH or LEH genotypes and the extent of aganglionosis for this locus.

We sequenced the coding regions and part of the non-coding regions of these three candidate genes. Then, we detected potentially causative genes that result in the phenotype difference between AGH and LEH strains. The RET ligand *Gdnf* is a known causative gene of HSCR and possibly interacts with *Ednrb*, the gene that modifies the aganglionosis phenotype [36]. Thus, we completely analyzed *Gdnf*. Two single-base mutations in the promoter region and seven mutations in the intron of *Gdnf* were found (Table 3). We evaluated the effects of *Gdnf* on the severity of aganglionosis through PCR-RFLP-based genotyping and correlation analysis with the ratio of the severity of aganglionosis. Considering the limited distance of these mutations, we selected g.76897291C > T in this procedure. As shown in Fig. 4A, AGH, LEH, and LL homozygotes were labeled as AA, LL, and AL, respectively. The AA genotype cannot be recognized by the restriction enzyme *Sty* I and showed one band (595 bp). The LL genotype can be completely divided into two bands (186 and 409 bp). The AL genotype showed three bands as expected. The results of correlation analysis revealed a significant difference between LL and AL, and a highly significant difference between AA and AL (Fig. 4B). This result indicates that this locus is closely related to the severity of aganglionosis.

**Table 3:** List of mutations in *Gdnf*, *Ptger4*, and *Slc45a2*.

Genes	Location	Sequence ofF344/LEH/AGH	Description
*Gdnf*	g.76896910, promoterg	C/C/T	Single-base transition in AGH
	g.76897291, promoter	C/C/T	Single-base transition in AGH
	g.76901040-76901042, intron 1	TTA/TTA/---	Deletion in AGH
	g.76901607, intron 1	G/G/A	Dingle-base transition in AGH
	g.76901863, intron 1	-/G/-	Insertion in LEH
	g.76918403-76918405, intron 2	AAG/AAG/---	Deletion in AGH
	g.76919183, intron 2	C/C/T	Single-base transition in AGH
	g.76919529, intron 2	G/A/G	Single-base transition in LEH
	g.76919749, intron 2	C/C/T	Single-base transition in AGH
*Ptger4*	g.73986958, promoter	G/G/A	Single-base transition in AGH
	g.73985633, exon 1	T/T/C	Synonymous mutation in AGH
*Slc45a2*	g.83718133, promoter	G/G/A	Single-base transition in AGH
	g.83718063, promoter	G/G/A	Single-base transition in AGH
	g.83717975, promoter	A/A/-	Deletion in AGH
	g.83717367, exon 1	A/A/T	Amino acid substitution in AGH
	g.83717275, exon 1	C/C/T	Synonymous substitution in AGH
	g.83715441, exon 2	G/G/A	Synonymous substitution in AGH

We also identified the mutation g.83717367 A > T in exon 1 of *Slc45a2*. This mutation results in a Thr32-to-Ser (T32S) substitution, which may alter the function of the corresponding protein and further affect the phenotype. Other mutations detected in *Slc45a2* and *Ptger4* are shown in Table 3.

## Discussion

HSCR is a multigenic disorder whose genetics is highly complex and shows unconformity with Mendel’s law. The variable phenotype and incomplete penetrance of HSCR suggest the involvement of modifier genes. Many studies focused on the interactions between known HSCR-associated genes that significantly influence the incidence and severity of intestinal aganglionosis. Genetic interactions of HSCR were first reported in a human study on the genetically isolated Mennonite population, and results suggest that *RET* and *EDNRB* interact to cause HSCR [14]. Subsequent studies found interactions between *PAX3* and *RET* [18], *PHOX2B* and *RET* [28], *SOX10* and *ZFBX1B* [29], *SOX10* and *EDN3* or *EDNRB* [30], and *RET* and *NRG1* [31]. Interfamilial variation and incomplete penetrance are commonly observed in HSCR. This observation strongly suggests that modifier genes are involved in the formation of intestinal aganglionosis. Modifier loci or modifier genes acting as protectors can affect the disease to some extent but cannot completely eliminate it [32]. Only a few modifier genes have been detected for HSCR to date. Clinical studies on human suggested that the X-linked gene *L1CAM* may be a modifier gene for *RET* [33]. *L1cam* can also modify the function of *Sox10* in mice [34] and *Edn3*/*Ednrb* [35] during the development of the enteric nervous system. A genome-wide scan in mice suggested that multiple modifier intervals are correlated with the severity of aganglionosis in Sox10 (Dom) mice and provided additional evidence of the multi-genic effects that contribute to aganglionic megacolon [36].

Genetic background modulates the severity of aganglionosis in animals [3, 22]. In our previous study, we established three rat strains carrying *Ednrb* mutations: AGH-*Ednrb* ^*sl*^, LEH-*Ednrb* ^*sl*^, and F344-*Ednrb* ^*sl*^. AGH-*Ednrb* ^*sl/sl*^ rats showed the most severe aganglionosis, followed by LEH-*Ednrb* ^*sl/sl*^ rats, and then F344-*Ednrb* ^*sl/sl*^ rats. We also detected a QTL on Chr 2 using an F_2_ intercross between AGH and F344 strains. Considering the varying severity degrees of aganglionosis among these three strains, we hypothesized that different strains contain different modifier(s) that influence the length of the affected gut. Therefore, we used the same method to explore the modifier(s) associated with the severity of aganglionosis using another F_2_ intercross between AGH and LEH strains. Interestingly, a QTL significantly associated with aganglionosis was detected in the Chr 2 region, and this QTL overlapped with the previously identified QTL [23]. This result suggests that this region contains a modifier that is related to HSCR. In our previous research, we found no mutations in the exons of the candidate gene *Gdnf* between the AGH and F344 strains [23]. Data from mouse studies showed that *Gdnf* is essential for the development of enteric neurons during embryogenesis [37, 38]. In the present study, we found two single-base mutations in the promoter region and seven mutations in the intron through direct sequencing, seven of which were common between AGH and F344 as well as between AGH and LEH (Table 3). The one within the promoter region may be included in the binding site of the transcription factor MNF, which refers to human data [39]. This mutation may change the expression level of *Gdnf* to modify the severity of aganglionosis. To evaluate the association of this mutation with the severity of aganglionosis, we performed a correlation analysis by genotyping the SNP of *Gdnf*. As expected, a high correlation was found between this mutation and the aganglionosis phenotype. This finding suggests that the mutation in the promoter may modify the severity of aganglionosis to some extent. However, other biological technologies, such as luciferase reporter assays and transgenic animals, should be employed for verification. Considering that *Gdnf* owns a multiple promoter system [39], we cannot exclude the possibility that other mutations in introns influence the expression of *Gdnf*. We compared known HSCR genes in humans and mice with candidate genes in the intervals of the modifiers mapped (S1 Table). Our results showed that *Gdnf* and *EDNRB* possibly interact to modify HSCR on the basis of the known interactions between the *Gdnf/Ret* and *Et3/Ednrb* signaling pathways [14]. However, further tests and studies need to be conducted for verification. Moreover, an unknown gene within this region possibly interacts with *Ednrb* to modify the length of the influenced gut.

We also identified that an A-to-T transition in exon 1 of *Slc45a2* results in a Thr32-to-Ser (T32S) substitution in the corresponding protein. Solute carrier family 45, member 2 (*Slc45a2*), known as membrane-associated transporter protein (MATP) or melanoma antigen AIM1, contains seven exons spanning a region of approximately 40 kbp. Many studies suggested that *Slc45a2* is related to oculocutaneous albinism type IV [40–42]. Du and Fisher [43] determined that *Slc45a2* is transcriptionally modulated by the melanocyte-specific transcription factor MITF. However, no reports have analyzed the relation of the enteric nervous system or HSCR to *Slc45a2*. In the present study, we investigated the effects of the mutations on the protein structure (amino acid substitution). Predictions obtained from Polyphen-2 (Score = 0,658) showed that the mutations can damage the human ortholog. Therefore, *Slc45a2* possibly affects the development of melanophores and enteric nervous system cells. Further research is needed to test this hypothesis and assess the effects of other mutations in *Slc45a2* and *Ptger4*.

## Materials and Methods

### Animals

Aganglionosis rats have a spontaneous *Ednrb* ^*sl*^ mutation. More than 20 generations have passed to establish the AGH inbred line carrying the *Ednrb* ^*sl*^ mutation. This mutation was crossed into another strain (Long–Evans) with a different genetic background for more than 10 generations to build the LEH-*Ednrb* ^*sl*^ (LEH) strain. Heterozygous AGH-*Ednrb* ^*sl*^ (n = 2) and LEH-*Ednrb* ^*sl*^ (n = 8) rats were bred to generate F_1_ animals, and then heterozygous male (n = 5) and female (n = 20) F_1_ progenies were used to produce F_2_ intercross (n = 592). A total of 149 *Ednrb* ^*sl/sl*^ progenies were selected on the basis of skin pigmentation patterns.That is, homozygous F_2_ mutant rats (*sl*) had almost no pigmentation on their heads compared with other genotypes of rats previously described [23]. To examine whether or not the method is correct, 215 F_2_ progenies (*Ednrb* ^*sl/sl*^, n = 50; *Ednrb* ^*+/sl*^, n = 108; *Ednrb* ^*+/+*^, n = 5 7) were genotyped by the PCR method with special primers (F-CCTCCTGGACTAGAGGTTCC and R-ACGACTTAGAAAGCTACACT), flanking the site of the 301 bp deletion. PCR products were electrophoresed in 1.5% agarose gels to distinguish the wild (511 bp) and mutated (210 bp) alleles. The genotyping results were consistent with the coat color method and showed that homozygous F_2_ mutant rats (*sl*) can be selected from F_2_ progenies on the basis of skin pigmentation. AGH (n = 35), LEH (n = 34), and F_1_ (n = 32) were raised to determine the severity of aganglionosis in each strain. The animals were maintained in specific pathogen-free conditions with feeding and drinking allowed ad libitum. The rats were maintained in a room under the following conditions: 22 ± 4°C temperature, 40%–60% relative humidity, and 12 h light–dark cycle. The rats were sacrificed by CO_2_ inhalation.

### Ethical Statement

All research and experimental protocols were approved by the Regulation for the Care and Use of Laboratory Animals, Hokkaido University (approval ID: No. 110226) and performed under the guidance of the Institute for Laboratory Animal Research (ILAR). All animals were housed in a facility approved by the American Association for Accreditation of Laboratory Animal Care International.

### Genotype analysis

A total of 149 F_2_ intercross of *Ednrb* ^*sl/sl*^ was selected for the genome-wide scan. Genomic DNA was extracted from tail clips using a standard phenol/chloroform method. All DNA samples were diluted to a standard concentration of 20 ng/μL. Ninety-two polymorphic microsatellite markers (Table 4) spanning 20 autosomes were genotyped across these intercross progenies. The map positions of the microsatellite markers were based on information from the Rat Genome Informatics (http://www.ncbi.nlm.nih.gov/probe, RGSC Genome Assembly v3.4). PCR of these polymorphic microsatellite markers was performed at 95°C for 5 min (one cycle), followed by 35 cycles of denaturation at 95°C for 30 s, primer annealing at 55°C for 30 s, and extension at 72°C for 30 s. All amplicons were electrophoresed in 10% acrylamide gels, stained with ethidium bromide (5 × 10^−9^ μg/mL) for 8 min, and then photographed under an ultraviolet lamp.

**Table 4:** Microsatellite markers used for genotyping the F_2_ intercross of *Ednrb* ^*sl/sl*^.

*Microsatellite Marker*	Position(Mbp)	*Microsatellite Marker*	Position(Mbp)	*Microsatellite Marker*	Position(Mbp)	*Microsatellite Marker*	Position(Mbp)
*D1Rat392*	21.6	*D4Rat183*	187	*D9Rat153*	107	*D14Rat94*	88
*D1Rat343*	98	*D4Rat204*	243	*D10Mgh27*	1.6	*D15Rat5*	25
*D1Rat269*	133	*D5Rat125*	22	*D10Rat217*	17	*D15Rat6*	37
*D1Rat159*	218	*D5Rat196*	107	*D10Rat177*	29	*D15Rat48*	66
*D1Got225*	255	*D5Rat44*	162	*D10Got60*	40	*D16Rat78*	21
*D2Rat252*	42	*D6Got15*	30	*D10Rat163*	50	*D16Rat3*	44
*D2Rat116*	52	*D6Got74*	71	*D10Mit2*	65	*D16Got63*	69
*D2Mgh14*	62	*D6Rat165*	103	*D10Rat154*	75	*D16Rat55*	78
*D2Rat201*	69	*D6Rat11*	124	*D10Rat7*	104	*D17Rat2*	68
*D2Mit33*	73	*D7Rat31*	32	*D11Got45*	67	*D17Rat12*	29
*D2Rat385*	79	*D7Got23*	36	*D11Rat63*	72	*D17Rat24*	50
*D2Mit5*	86	*D7Got36*	47	*D11Rat43*	90	*D17Rat175*	87
*D2Rat21*	95	*D7Rat73*	61	*D12Rat58*	0.5	*D18Rat132*	26
*D2Rat123*	132	*D7Rat143*	105	*D12Rat76*	34	*D18Rat34*	48
*D2Got114*	191	*D7Rat131*	115	*D12Rat14*	34	*D18Got63*	68
*D2Rat241*	243	*D8Rat68*	21	*D12Rat86*	45	*D18Rat86*	66
*D3Rat57*	8	*D8Rat33*	79	*D13Rat150*	20.8	*D19Rat15*	27
*D3Mgh7*	45	*D8Rat18*	99	*D13Rat149*	50	*D19Rat27*	30
*D3Rat34*	89	*D8Rat8*	121	*D13Rat180*	67	*D19Got53*	62
*D3Rat287*	111	*D9Got6*	4	*D13Rat131*	88	*D20Mit4*	34
*D3Rat78*	159	*D9Rat41*	14	*D14Got35*	29	*D20Rat55*	46
*D4Mgh16*	61	*D9Got27*	20	*D14Mit4*	44	*D20Got47*	52
*D4Rat26*	135	*D9Mit3*	63	*D14Rat45*	88		

For SNP genotyping, PCR-RFLP analysis was performed with the restriction enzyme *Sty* I (recognition sequence: …5′-C↓CWWGG-3′…). Specific primers (F: 5′-CGTGGTGTCTCGTTCGGA-3′; R: 5′-CCGCTTGCCTTCCTACTT-3′) were designed for PCR, the product length of which was 595 bp. Digestion reaction was performed following the manufacturer’s recommended protocol. The digested products were detected using 1.5% agarose gels.

### AChE staining

The guts from the proximal esophagus to the end of the colon were dissected as a single piece for rats at 14 d postnatal. Attachments were removed before the guts were processed using AChE whole-mount staining [16]. The enteric ganglia were visualized under a microscope to determine the extent of the affected gut by aganglionosis. The lengths of the aganglionic gut and the large intestine (from the cecum to the anus) were measured. The ratio of these two lengths was calculated to indicate the severity of aganglionosis.

### Linkage analysis

Genotyping data combining the ratio of aganglionosis extent were analyzed by Map Manager QTXb20 software. This software uses a maximum likelihood algorithm with “interval mapping” and “simultaneous search,” and permits efficient localization of loci. Recombination frequencies (%) were converted into genetic distance (cM) using the Kosambi map function. This program provides linkage data as an LRS score. Genome-wide significance thresholds were calculated in terms of LRS by carrying out 1000 permutations based on the established guidelines. The thresholds in the intercross progenies were determined by QTL software for finding suggestive, significant, and highly significant linkages.

### SNP screening for candidate genes

The coding regions and part of the non-coding regions of the candidate genes *Gdnf*, *Ptger4*, and *Slc45a2* were amplified on the basis of the reference sequence (NC_005101.3) by using the genomic DNA of AGH and LEH rats. Primers (S2 Table) were designed using Primer Premier 5.0 software. PCR products were directly sequenced by an ABI PRIZM 377 DNA sequencer (Perkin-Elmer). DNA sequences were analyzed using the DNASTAR 5.0 package (DNAstar, Madison, Wis., USA).

### Statistical analysis

To evaluate the effect of the mutation found in the upstream region on the severity of aganglionosis, the mean values for data sets were compared by one-way ANOVA followed by post-hoc test.

## Supporting Information

S1 Table Interactions between known HSCR associated genes and their modifiers. (PDF)

S2 Table Primers information for *Ptger4*, *GDNF* and *Slc45a2*. (PDF)
