# Supplementary material for: Identification of Genetic Loci Affecting the Severity of Symptoms of Hirschsprung Disease in Rats Carrying Ednrbsl Mutations by Quantitative Trait Locus Analysis
Source: PLoS One. 2015 Mar 19;10(3):e0122068. doi: 10.1371/journal.pone.0122068 (PMC4366197; doi:10.1371/journal.pone.0122068)
Supplement: S1 Table — (PDF) [file pone.0122068.s001.pdf]

Gene	Interaction
RET	*Phox2b*, *Sox10*, *Ednrb/Et-3*, *NRG1*, *L1cam*, *BBS 4 5 7*, *3q21*, *9q31*, *4q31-32*, *19q12*
Sox10	*RET*, *Ednrb/Et-3*, *ZFHX1B*, *L1cam*, *sox8*, *Mouse Chr 4 8 11*
Ednrb/Et-3	*RET*, *Sox10*, *L1cam*

Red: HSCR associated gene; Blue: known HSCR modifier gene; Black: Modifier loci with unknown gene.

参考文献： Adam S Wallace, Richard B Anderson. Genetic interactions and modifier genes in Hirschsprung's disease. *World J Gastroenterol* 2011 December 7; 17(45): 4937-4944
