# Supplementary material for: Identification of Genetic Loci Affecting the Severity of Symptoms of Hirschsprung Disease in Rats Carrying Ednrbsl Mutations by Quantitative Trait Locus Analysis
Source: PLoS One. 2015 Mar 19;10(3):e0122068. doi: 10.1371/journal.pone.0122068 (PMC4366197; doi:10.1371/journal.pone.0122068)
Supplement: S2 Table — (PDF) [file pone.0122068.s002.pdf]

Primer pair	Primers（5’→3’）	Annealing temperature	Products length（bp）	Amplified region
Ptger4-P1	F: GGCAACGCCTGGAAAAATC<br>R: GTCGGGACGATTGCTTTAG	54℃	518	Promoter
Ptger4-P2	F:TCTCCGCTCTGTGACTCC<br>R: AAGGCTGCCAACTTTCCG	54℃	870	Promoter
Ptger4-P3	F: ACCTACGCTCCGTTCCA<br>R: CTGTTCTTTGGCTTCCCG	54℃	424	Promoter
Ptger4-P4	F: GTTCCGAGAGCGGCAAAT<br>R: GCTGGGCTTACCAACAATG	56℃	834	Promoter
Ptger4-P5	F: GCAAGTTGTAAATGGCTC<br>R: GCTGTAGAAGTAGGCGT	50℃	481	Exon 1
Ptger4-P6	F: TGTCGGGTCTCAGCATCAT<br>R: AGGAAAAACCTGGGTCTGC	56℃	725	Exon 1
Ptger4-P7	F: CGGTAAAGCATAAGCAAC<br>R: AGACAGCCCTTCTGAGTA	50℃	919	Exon 2
GDNF-P1	F: CCGTCACCAAGTTGCGATT<br>R: TCTCCGTAGACCCCCAGTT	60℃	822	Promoter
GDNF-P2	F: CGTGGTGTCTCGTTCGGA<br>R: CCGCTTGCCTTCCTACTT	54℃	595	Promoter and exon1
GDNF-P3	F: CGAGGTAAGAGGGGAAACG<br>R: GAAGGGGAGGAATGCCG	58℃	526	Intron 1
GDNF-P4	F: GGTGCTGAGAACCTAAAAAT<br>R: CAGGGCAAGAAAGAAACA	59℃	692	Intron 1
GDNF-P5	F: ATGCGACCAACCCGTAAT<br>R: CATAAAACAGTAGGGGAACC	54℃	751	Intron 1
GDNF-P6	F: GCCCGAAAAGCGAGGATT<br>R:GAGGAGAAGCAGGAGAAACG	59℃	655	Intron1
GDNF-P7	F: ACGCACTCCCCATCTTTG<br>R: TTCCAGTTTGGCTCACGG	58℃	282	Intron 1
GDNF-P8	F: TAAAAGCCTTCCGTGAGC<br>R: CATCCCTTGGGCGTCTGTT	59℃	432	Intron1
GDNF-P9	F: GTTCTGTCGCTTCCCAATC<br>R: ACACCCGAAAATGCCCTT	58℃	687	Intron 1
GDNF-P10	F: CCAAAGGACTTAGTGTTCAGGA<br>R: ACACCCCACATTTCCTCAG	54℃	660	Intron 1
GDNF-P11	F: CTGTTGTTCGCATAGGTG<br>R: CCATTTAGCCCGAGAGTG	58℃	800	Intron 1 and exon 2
GDNF-P12	F: AGGATGAGCCACCCCAAC<br>R: TCCCTTACCCACCTCCATC	56℃	578	Intron 2
GDNF-P13	F: TACCCCATACTTGCTCACG<br>R: GCAGACCAGGGAAGATGAA	56℃	790	Intron 2
GDNF-P14	F: TATTCAGACTCTCGGGTG<br>R: CCTTGATGCTCTGGTTTG	54℃	539	Intron 2

GDNF-P15	F: GATACCCACACATACCAG<br>R: GTCAGGATAATCTTCGG	54℃	520	Intron 2
GDNF-P16	F: TCTTAGAAACCTGTCTCCCG<br>R: AGGGTCAGATACATCCACAC	54℃	738	Exon 3
Slc45a2-P1	F: CCATTGGCGTTCTTACACC<br>R: CATTGGCGTTCTTACACC	52℃	630	Promoter
Slc45a2-P2	F: AAGATTTTCCCTGCCTG<br>R: GGACTACATTTCCCAGAC	54℃	514	Promoter
Slc45a2-P3	F: TGCCCCTGGCTTTTCAC<br>R: GGTCCTTAGAATGCTCGC	54℃	806	Promoter
Slc45a2-P4	F: GCGAGCATTCTAAGGAC<br>R: GCAGTGTCGTGATGGTTC	54℃	802	Promoter and exon 1
Slc45a2-P5	F: CAAAAGCAAGGCGGTTCG<br>R: TCAGGAACTCTGTAGCCCCA	54℃	478	exon 2
Slc45a2-P6	F: CAGTCAGTCTCTGGTCTCTA<br>R: GGACAAGCAAATACGACAC	54℃	611	exon 3
Slc45a2-P7	F: CATTCATTTCACACAGCAC<br>R: CGCAGTGTTTCATTTTAC	50℃	429	exon 4
Slc45a2-P8	F: CTCAAGGGTAGAGAAGCAT<br>R: GGTTTTTTTCCCCGTCT	50℃	332	exon 5
Slc45a2-P9	F: TATTGTGAGGCACTACCAG<br>R: TATTTGGGGGTCACTATC	50℃	433	exon 6
Slc45a2-P10	F: TAAGGCTGCGTGTGAATG<br>R: GTCCGAGAAAAGAGCGATT	54℃	469	exon 7
